# A Systematic Review of the Effectiveness of Intergenerational Programs

**DOI:** 10.3389/fpsyg.2017.01882

**Published:** 2017-10-27

**Authors:** Alejandro Canedo-García, Jesús-Nicasio García-Sánchez, Deilis-Ivonne Pacheco-Sanz

**Affiliations:** ^1^Department of Psychology, Sociology and Philosophy, University of León, León, Spain; ^2^Department of Psychology, University of Valladolid, Valladolid, Spain

**Keywords:** intergenerational program, intervention, research, evaluation, evidence-based practices

## Abstract

**Purpose of the study:** The objective of the present review study is to identify the determinant elements of the effectiveness of empirically based interventions (EBI) in the field of intergenerational work, contrasting face-to-face and combined (face-to-face and virtual) intervention modalities against variables relating to this field according to EBI indicators.

**Design and Methods:** An extensive literature search returned a total of 553 studies. Of these, just 50 studies met the inclusion criteria of being an empirical investigation of the effectiveness of intergenerational programs that contain appropriate elaboration on theoretical constructs and methods.

**Results:** The descriptive and multivariate analysis conducted demonstrates that programs with a greater number of EBI controls have the greatest effectiveness, regardless of the intervention mode employed, and that this effectiveness is also modulated by other variables such as the participants' disabilities, their literacy level, or their membership of an organization.

**Conclusions:** We examined the implications of these findings, noting the need to increase the number of virtual interventions that could improve the efficiency of the activities undertaken, and at the same time ensuring that EBI indicators are also fulfilled.

## Background and objectives

Published reviews of the literature on intergenerational programs have shown how these interventions promote satisfaction and quality of life in all the parties involved (Kuehne, 2003). Indeed, priorities among the European Union is the promotion, through different initiatives and public organisms, of collaborative learning between generations (European Commission, 2012; ECIL, 2013)—that is, the so-called intergenerational programs, which is understood as the activities that foster cooperation, interaction, and exchange between two or more generations (Kaplan and Sánchez, 2014). These are appropriate complements for lifelong learning among older adults and for the development of a change in the attitudes that young people exhibit toward the elderly (Borrero, 2015; Park, 2015; Thompson and Weaver, 2016). As proven by various authors, participation in interventions of this type yields benefits in terms of improving older adults' health and well-being by facilitating continued intellectual or physical activity in the elderly, and it simultaneously contributes to the encouraging of values and behaviors in children and to the construction of identity among adolescents (Celdrán et al., 2009; Galbraith et al., 2015; Fujiwara, 2016; Sakurai et al., 2016).

Nevertheless, although intergenerational programming in education, aging services, and mental health is a popular idea that is currently being promoted, evidence of the effectiveness of these programs is limited. Many of them present only anecdotal evidence of impact, limited use of theory and standardized measures, assessment of only one generation of participants, and an absence of longitudinal evaluations (Jarrott, 2011).

To all these drawbacks, we must add the fact that face-to-face interventions are still prevalent in this field, which is a tendency that often overlooks the benefits of technology in promoting contact between generations (Fricke et al., 2013). For instance, despite the difficulties involved in using digital media with older people, the benefits these programs could offer them are optimum due to their attitude towards them (Aarts et al., 2015; Heo et al., 2015). In addition, recent studies in other areas, such as that by Andersson et al. (2016), reveal comparable effectiveness, which is understood as the level at which the activities of a program produce the desired effect, between face-to-face and virtual modes of intervention. Together with the possibility that virtual interventions achieve a higher degree of efficiency (regarded as the cost of producing products or services relative to other programs or to some ideal process) by reducing the staff time and money required to a minimum, this finding of comparable effectiveness could influence the future practice and determine the results of these activities. In this regard, it seems appropriate to consider these issues in relation to the field of intergenerational work, where programs are aimed at closing the digital divide between the older and younger generations that already exists (Wu et al., 2016).

In general, a core question in relation to the different programs concerns the related matter of an evaluation study's methodological rigor, which includes the clarity of the intervention's definition, its dosing or timing, the rigor of measurement and fidelity to treatment, etc. Such considerations justify the need to develop a greater amount of *evidence-based programs* (EBP) within the intergenerational field, which “reflect a translation of testable research theories into key intervention elements that resonate with program adopters and intended participants” (Ory and Smith, 2015, p. 1). This process has been carried out in many studies (Vaughn et al., 2011; Stirman et al., 2013, 2015; Troia and Olinghouse, 2013; Campbell et al., 2014; Troia et al., 2015; CEEDAR, 2016a,b; Ciullo et al., 2016; Gunn and Delafield-Butt, 2016) in the field of *learning and developmental disabilities* (LDD) and in others besides.

This line of research would involve evaluating *empirically based interventions* (EBI), which are the focal object of analysis in social and scientific discourses on healthy aging (Sadana et al., 2016) and in the application of the principles from the life-cycle approach to research on aging (Díaz and García, 2016). However, EBI have scarcely been examined within the intergenerational context.

Accordingly, the objective of the present review is to identify the relevant elements that ensure the effectiveness of empirically based intergenerational interventions, contrasting face-to-face, virtual, and combined (face-to-face and virtual) intervention modalities against variables relating to the field of intergenerational work according to EBI indicators. In relation to the *effectiveness* of the programs, we hypothesize: (a) the non-existence of statistically significant differences according to the intervention modality used; (b) its dependence on the maximum fulfillment of EBI indicators; and (c) the moderating influence that other variables such as participants' disorders exercise on this.

## Design and methods

### Selecting the studies

We conducted our search in 2015. Our language scope included English. In order “to avoid the biased retrieval of searching only the major journals, which may selectively publish only the results characterized by lower *p* values and larger effect sizes” (Rosenthal, 1995, p. 184), in this systematic review, we used some of the techniques recommended by Cooper and Hedges (1994) and Cooper (2009) such as: (a) direct-to-researcher channels (personal contact and mass solicitation), (b) quality control search techniques (peer-reviewed journals), and (c) secondary searching techniques (the Internet, reference databases, and citation indexes). On this basis, we mined the Web of Science, PsycINFO, ERIC, and Google Scholar databases for peer-reviewed articles published between 2004 and 2015 using the following keywords**:** *intergenerational, program, effectiveness, research*, and *evaluation*.

Table 1 presents a log that has been used to keep track of the techniques used to search the literature, based on the one developed by Cooper (2009).

**Table 1:** A log for keeping track of a search of the literature (adapted from Cooper, 2009).

**Direct-to-researcher search techniques**	**Who was contacted**	**Date sent**	**Date reply received**	**Nature of reply**
Personal contact	S. J.	11/06/2015	11/06/2015	Sent 2 articles plus contacts
	J. C.	12/06/2015	12/06/2015	Sent 1 article
	T. K.	12/06/2015	13/06/2015	Sent 1 article
	D. G.	12/06/2015	13/06/2015	Sent 2 articles
	R. K.	12/06/2015	15/06/2015	Sent links for 3 articles
	R. Z.	20/06/2015	20/06/2015	Sent 3 articles
	D. V.	20/06/2015	20/06/2015	Sent 1 article
	F. V.	20/06/2015	21/06/2015	Sent 5 articles
	A. S.	28/06/2015	28/06/2015	Sent 1 article
	M. F.	28/06/2015	06/07/2015	Sent 1 article
	Y. M.	28/06/2015	09/07/2015	Sent 2 articles
Mass solicitation	University of León	08/06/2015	22/06/2015	Sent 72 articles
Quality-controlled search techniques	Organization names or journal titles	Years Searched	No. of documents examined	No. of relevant documents found
Peer-reviewed journals	*Journal of Intergenerational Relationships*	2004-2015	396	12
	*Educational Gerontology*	2004-2015	767	13
Secondary searching techniques	Search engines/Database names/Index names	Years covered	Search procedure	No. of documents found
Internet	scholar.google.com	2004-2015	“Intergenerational programs” effectiveness research	301
Reference databases	PsycINFO	2004-2015	(Intergenerational programs) AND effectiveness AND research	19
	ERIC (via EBSCOhost)	2004-2015	(SU intergenerational programs) AND (SU effectiveness) AND (SU research)	132
Citation indexes	Web of science	2004-2015	TS = (intergenerational programs) AND (effectiveness) AND (research)	27

*Source. Compiled by the authors*.

### Inclusion and exclusion criteria

In general, the criteria followed in order to guarantee the methodological quality of the studies selected was based on The PRISMA Statement (Moher et al., 2009), an evidence-based minimum set of items for reporting in systematic reviews and meta-analyses.

Initially, 601 articles were obtained from the search. After removing the duplicates, we were left with a total of 553 articles. We read the abstracts of all the papers and narrowed the corpus to 284 studies according to the following inclusion criterion: the publications needed to be an empirical investigation of the effectiveness of intergenerational programs. That is, they needed to establish the effectiveness of a program through a large, carefully controlled experimental research study involving hundreds of subjects who are randomly assigned to experimental and control (or comparison) groups.

Then, after a deeper screening based on the whole text, we excluded 234 works that contained insufficient elaboration on theoretical constructs or methods, as this characteristic hindered inferences regarding the results' contribution to understanding intergenerational phenomena related to the benefits obtained by the participants.

**Figure 1:**
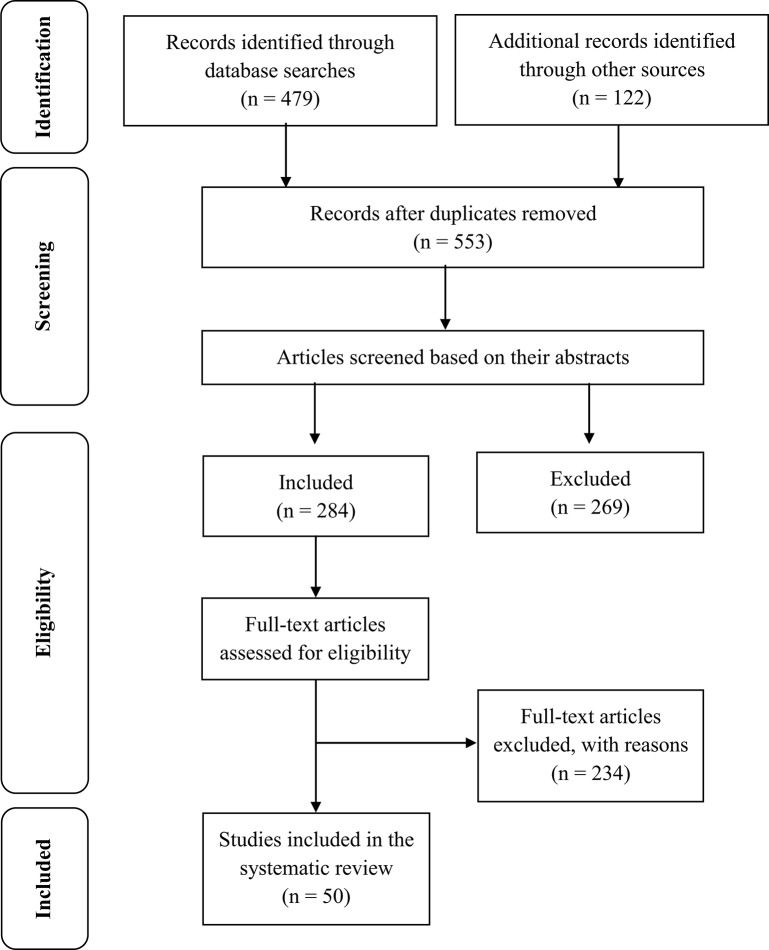
PRISMA flowchart of study selection. PRISMA, Preferred Reporting Items for Systematic Reviews and Meta-Analyses. *Source*. Based on Moher et al. (2009).

Thus, a total of 50 empirical studies were identified that met the inclusion criteria. A flow diagram (Figure 1), adapted from the one established by Moher et al. (2009), reports information on these phases of the review process.

All papers were abstracted into a shared spreadsheet. The studies were reviewed and coded by all of this paper's authors to assure inter-rater reliability for inclusion.

### Coding the studies

To code the information detailed in the 50 articles, we used a coding instrument that focused on a total of 50 variables, which were classified into three sections that referred to:

#### The general focus or quality-of-life dimension addressed by the study

The different outcomes related to quality of life worked on in the intergenerational intervention programs reviewed were classified within their respective dimensions of quality of life in accordance with those established by Verdugo Alonso et al. (2013): *self-determination (Seld), emotional well-being (EmW), physical well-being (PhW), material well-being (MatW), personal development* (*PersDel), social inclusion (SocIncl)*, and *interpersonal relations (InterRel)*.

#### The characteristics of the reviewed study

This section includes:

- The general characteristics of the studies and interventions: year, field of knowledge (gerontology, health sciences, and education), country, intervention modality (face-to-face, virtual, or combined), number of virtual resources, intervention context, duration, and type of evaluation instrument;
- The characteristics of the intergenerational groups and participants: sample size, sample attrition, number of control and experimental intergenerational groups, number of participants per control and experimental intergenerational groups, mean age of the intergenerational groups, classification of the mean age of intergenerational groups by age range (Martín, 2005), participants' disorder (mental, physical, or combined), gender, academic level, literacy level, digital competence, socio-communicative competence, professional interest, situation of risk of exclusion, psycho-social discomfort, physical capacities, mental capacities, membership of an organization, commitment to attending the program, and geographical proximity;
- Effect sizes (pre-test and post-test mean and standard deviation).

#### EBI indicators or controls

Recording of sessions, training of instructors, instruction of participants, definition of variables, intervention protocol, intervention modality contrast, generalization, pre-post measures, follow-ups, and total indicators.

### Calculating the effect size

Once the coded recording of the data that the previously described variables refer to had been completed using *Excel* matrices, we calculated the effect sizes using Cohen's *d*, interpreting these based on Rosenthal's (1996) expanded classification, in which those between 0 and 0.29 are considered Small (Sma); those between 0.30 and 0.79 as Medium (Med); those between 0.80 and 1.29 as Large (Lar); and those with a magnitude of 1.30 or greater as Very Large (VerLar). Cohen's *d* was calculated by subtracting the mean of the control group from the mean of the intervention group and dividing the obtained difference by the averaged standard deviations for the intervention and control groups (Cohen, 1988); that said, in the 17 interventions that have a quasi-experimental design of a single experimental intergenerational group with pre and post measures, the effect size was calculated by subtracting the post-treatment mean from the pre-treatment one.

Descriptive and multivariate statistical analyses were carried out using SPSS 22.0. Multivariate analysis included general linear models (GLM), considering as grouping variables: the *effect size*, the *intervention modality*, the *total EBI indicators*, the *classification by age range*, the *quality of life dimension*, and the *disorder associated with the participants*.

## Results

### Description of the studies included in the review

#### Characteristics of the intergenerational groups and participants

The sample size is small (51 people or fewer) in 27 of the 50 studies analyzed; 19 studies have a medium sample size (52–200 people); and 4 have a large sample size (more than 200 people). Participants are predominantly female in 30 of the 50 selected articles; there is only one with the same number of users for both genders (Table 2).

**Table 2:** Characteristics of the intergenerational groups and participants.

**Study**	**Groups^a^**	**Sample^b^**	**Participants per group**^c^			***M (SD*) Age^d^**	**Age range^e^**	**Gender (%)**	
			**CG**	**EG1**	**EG2**			**Women**	**Men**
**FACE-TO-FACE MODALITY**									
Alcock et al., 2011	1	31	–	31	–	42	40-49	52.35	47.65
Barrowclough and White, 2011	1	20	–	20	–	29.75	21-39	70	30
Belgrave, 2011	2	50	21	29	–	–	–	–	–
Bernard et al., 2011	2	36	–	18	18	36.83	21–39	–	–
Boswell, 2012	1	43	–	43	–	20.98	15–20	90.7	9.3
Carson et al., 2011	1	20	–	20	–	44	40–49	83.3	16.7
Chippendale, 2013	1	11	–	11	–	56	50–59	63.3	36.7
Chippendale and Boltz, 2015	2	84	30	54	–	22.65 (4.9)	21–39	83.6	16.4
Cordella et al., 2012	1	260	-	260	–	45.08	40–49	55.15	44.85
DeMichelis et al., 2015	1	23	-	23	–	45 (3.93)	40–49	–	–
Fujiwara et al., 2009	2	141	74	67	–	68.45 (5.4)	60–69	73.27	26.73
Gaggioli et al., 2014	1	146	-	146	–	39.26 (3.75)	21–39	–	–
Gallagher and Carey, 2012	1	37	-	37	–	53.6	50–59	75.5	24.5
Gebbels et al., 2011	1	89	–	89	–	27.5	21–39	–	–
George and Singer, 2011	2	47	7	40	–	46.27	40–49	–	–
George, 2011	2	47	7	40	–	46.27	40–49	–	–
Heyman et al., 2011	2	50	18	32	–	4.51 (0.43)	Minors_6	45	55
Hsu et al., 2014	2	118	63	55	–	70.75 (6.87)	70–84	71.55	28.45
Isaki and Towle, 2015	1	16	–	16	–	44.75 (3.16)	40–49	54.13	45.87
Jackson et al. (2014)	1	28	–	28	–	45.1 (9.05)	40–49	–	100
Jarrott and Smith, 2011	2	59	–	35	24	31.25	21–39	42.5	57.5
Jarrott et al., 2011	2	40	–	18	22	35.5	21–39	98.3	1.7
Kamei et al., 2011	1	29	–	29	–	51.36 (5.76)	50–59	90.47	9.53
Karasik, 2005	2	80	–	51	29	20	15-20	72.90	27.1
Maddox et al., 2011	1	129	–	129	–	3.5	Menores_6	–	–
Mantie-Kozlowski and Smythe, 2014	1	2	–	2	–	34.5	21–39	50	50
McCleary, 2014	1	92	–	92	–	28.4	21–39	78.2	21.8
Murayama et al., 2015	2	136	82	54	–	69.1 (3.6)	60–69	83.8	16.3
Pinazo-Hernandis and Luna, 2011	2	20	6	14	–	47.5	40–49	100	–
Ransdell et al., 2003	2	40	–	20	20	30.22 (4.34)	21–39	100	–
Ransdell et al., 2004a,b	2	48	21	27	–	36.41 (3.29)	21–39	100	–
Ruggiano, 2012	2	9	–	5	4	50	50–59	-	–
Sánchez et al., 2011	1	306	–	306	–	53.6	50–59	80.2	19.8
Skropeta et al., 2014	1	139	–	139	–	43.25 (5.77)	40–49	–	–
Sterns et al., 2011	1	40	–	40	–	61.5 (7.52)	60–69	–	–
Strand et al., 2013	1	86	–	86	–	47	40–49	–	–
Suzuki et al., 2014	2	58	29	29	–	73.15 (6.25)	70–84	91.37	8.63
Tam, 2014	1	47	–	47	–	43.5 (2.98)	40–49	–	–
Vélez-Ortiz et al., 2012	1	17	–	17	–	55	50–59	88	12
Villar et al., 2013	1	286	–	286	–	52.2 (5.90)	50–59	82.15	17.85
Young and Janke, 2013	1	195	–	195	–	69.5	60–69	78	22
Zucchero, 2009	1	144	–	144	–	–	–	69.5	30.5
Zucchero, 2011	1	134	–	134	–	51.07 (5.28)	50–59	70.56	29.44
**COMBINED MODALITY**									
Chonody and Wang, 2013	1	26	–	26	–	75	70–84	73.07	26.93
Coppola et al., 2012	1	281	–	281	–	49.77 (7.98)	40–49	61.65	38.35
Council for Third Age, 2012	2	66	–	28	38	41.5	40–49	–	–
Gamliel and Gabay, 2014	1	61	–	61	–	41.5	40–49	–	–
George et al., 2011	2	47	7	40	–	46.27	40–49	–	–
Sanders et al., 2013	1	92	–	92	–	73 (9.6)	70–84	75.8	22.6
Shedletsky, 2012	1	33	–	33	–	46	40–49	74.42	25.573

*CG, control group; EG, experimental group; the SD is only included when it is available in the study or when the study allowed it to be calculated*.

a *Number of control and experimental intergenerational groups*.

b *Sample size*.

c *Number of participants per control and experimental intergenerational groups*.

d *Mean age of the intergenerational groups*.

e *Classification of the mean age of intergenerational groups by age range*.

*Source. Compiled by the authors*.

In terms of patients' disorders, mental ones are more prominent than physical and combined (mental and physical) for most studies. Articles also attend to participants' socio-communicative skills (40 of the 50 studies reviewed), their membership of an organization (35 cases), and the geographical proximity of the sample (36 of the 50 interventions examined).

#### Characteristics of the interventions and EBI controls

The dominant field of knowledge from which intergenerational interventions are carried out is gerontology, followed by health sciences and education studies. The United States, the country with most empirical studies published in relation to the subject of focus, provides a total of 30 to the present review, followed by Japan with 4, Spain, the United Kingdom, and Canada in third place with 3 studies each, China and Australia with 2, and Singapore, Italy, and Israel with 1.

In terms of intervention contexts, schools are the most widely used among a wide range that encompasses nursing homes, day centers, social and health centers, and even private homes. In addition, many of the interventions were carried out in multiple contexts.

The mean duration of the programs is 25 weeks (6 months), with variability in terms of the number and duration of sessions, whereas different types of self-reporting, and to a lesser extent interviews, systematic observation, and task resolution constitute the types of evaluation instruments that are most frequently used.

Finally, as can be seen in Table 3, which contains the number of studies analyzed that fulfill the different EBI indicators, we found a total of 43 works belonging to the face-to-face (Fac) intervention modality, against 7 from the combined (Comb) modality, in which sessions are conducted through a combination of traditional teaching and online learning. All of the 43 studies that involve the face-to-face intervention modality comply with empirically based indicators related to recording of sessions, definition of the variables, and intervention protocol. The 7 studies that involve the combined intervention mode entirely comply with up to 5 controls: recording of sessions, training of instructors, instruction of participants, definition of variables, and intervention protocol. It should be noted that only 15 studies on either method described the carrying out of a follow-up to the interventions by the programs.

**Table 3:** Fulfilment of EBI controls within studies included in the review.

**Modality**	***N***	**Recording of sessions**	**Training of instructors**	**Instruction of participants**	**Definition of variables**	**Intervention protocol**	**Intervention modality contrast**	**Generalization**	**Pre-post-measures**	**Follow-ups**
Face-to-face	43	43	42	39	43	43	3	6	30	14
Combined	7	7	7	7	7	7	2	1	6	1
Total	50	50	49	46	50	50	5	7	36	15

*Source. Compiled by the authors*.

Virtual studies discovered through the search process described above did not have the degree of empirical validity required to be included in this review.

### Comparative analysis between variables (GLM)

Multivariate analysis conducted via the general linear model shows statistically significant *multivariate contrasts* with large η^2^ effect sizes, for all grouping variables taken into consideration: *effect size* [λ_*Wilks*_ = 0.163; *F*_(160, 1, 178.586)_ = 4.240; *p* ≤ 0.001; η^2^ = 0.364]; *intervention modality* [λ_*Wilks*_ = 0.106; *F*_(36, 290)_ = 67.952; *p* ≤ 0.001; η^2^ = 0.894]; *total EBI indicators* [λ_*Wilks*_ = 0.001; *F*_(228, 1, 759.930)_ = 39.594; *p* ≤ 0.001; η^2^ = 0.834]; *classification by age range* [λ_*Wilks*_ = 0.001; *F*_(273, 2, 034.774)_ = 21.363; *p* ≤ 0.001; η^2^ = 0.736], *quality of life dimension* [λ_*Wilks*_ = 0.020; *F*_(216, 1, 769.607)_ = 7.631; *p* ≤ 0.001; η^2^ = 0.478], and *participants' disorder* [λ_*Wilks*_ = 0.002; *F*_(111, 896.457)_ = 62.574; *p* ≤ 0.001; η^2^ = 0.885].

We will now indicate the tests for inter-subject effects for each of the grouping variables and *post-hoc* contrasts for each dependent statistically significant variable.

#### According to effect size

It was demonstrated that the greater the sample attrition, the lower the effect size of the studies and vice versa [*M*_*Sma*_ = 5.3; *M*_*Med*_ = 5; *M*_*Lar*_ = 3.18; *M*_*VerLar*_ = 1.33; *F* = 3.34; *p* = 0.01; η^2^ = 0.04]; a high literacy level of individuals [*M*_*Sma*_ = 1.85; *M*_*Med*_ = 1.9; *M*_*Lar*_ = 1.93; *M*_*VerLar*_ = 1.93; *F* = 2.54; *p* = 0.04; η^2^ = 0.03] increases the effect of the programs on those individuals, which is also conditioned by the academic level of the users [*M*_*Sma*_ = 1.63; *M*_*Med*_ = 1.68; *M*_*Lar*_ = 1.7; *M*_*VerLar*_ = 1.7; *F* = 8.04; *p* = 0.001; η^2^ = 0.09]. In relation to the given disorder, it was also the case that as this intensified, the impact of interventions, which mainly addressed mental disorders, grew [*M*_*Sma*_ = 3.22; *M*_*Med*_ = 3.23; *M*_*Lar*_ = 3.73; *M*_*VerLar*_ = 3.74; *F* = 4.05; *p* = 0.003; η^2^ = 0.05]. In addition, membership of an organization corresponds to a greater effectiveness of the interventions [*M*_*Sma*_ = 1.4; *M*_*Med*_ = 1.42; *M*_*Lar*_ = 1.52; *M*_*VerLar*_ = 1.53; *F* = 2.71; *p* = 0.03; η^2^ = 0.03], which were undertaken with great success in the cases of people at risk of exclusion [*M*_*Sma*_ = 1.72; *M*_*Med*_ = 1.78; *M*_*Lar*_ = 1.89; *M*_*VerLar*_ = 2; *F* = 2.57; *p* = 0.04; η^2^ = 0.03]. Finally, a greater geographical proximity of the sample did not ensure a greater effectiveness of the interventions [*M*_*Sma*_ = 1.09; *M*_*Med*_ = 1.09; *M*_*Lar*_ = 1.07; *M*_*VerLar*_ = 1; *F* = 8.92; *p* = 0.001; η^2^ = 0.01].

On the other hand, a collation of the *post-hoc contrasts* does not reveal statistically significant differences based on the intervention modality employed; this was demonstrated instead in relation to fulfillment of the maximum number of EBI indicators (*p* ≤ 0.05), as well as the recording of sessions (*p* ≤ 0.04), and the follow-up of interventions (*p* ≤ 0.03), for small vs. large effect sizes. In total, statistically significant differences were found in 44 of the 216 analyzed contrasts (20.37%).

#### According to the intervention modality

We did not find significant differences in relation to the fulfillment of EBI indicators or based on the effect between face-to-face vs. combined interventions. However, we did find significant differences based on the fields of study through which the interventions were carried out [*M*_*Fac*_ = 2.8; *M*_*Comb*_ = 2.3; *F* = 4.71; *p* = 0.03; η^2^ = 0.01], in relation to the sample size (which was greater in the combined intervention modality) [*M*_*Fac*_ = 1.46; *M*_*Comb*_ = 1.85; *F* = 6.06; *p* = 0.01*;* η^2^ = 0.02] and to the level of socio-communicative competence (which was higher in the face-to-face intervention modality) [*M*_*Fac*_ = 1.37; *M*_*Comb*_ = 1; *F* = 5.9; *p* = 0.02; η^2^ = 0.02]. Gerontology, health sciences, and education intergenerational programs predominantly used a face-to-face modality.

#### According to the total EBI indicators

We found statistically significant results for the majority of the variables analyzed. The most notable results are those that demonstrated a relationship wherein the greater the number of EBI indicators fulfilled by the study, the lower the level of socio-communicative competence and the lower the physical and mental capacities of the participants (Table 4). Similarly, psycho-social discomfort on the part of users positively influences the quantity of indicators developed, thus additionally demonstrating that the employment of a greater number of virtual resources is not proportional to the accomplishment of the maximum number of EBI indicators.

**Table 4:** Statistically significant results taking as grouping variable the total number of EBI indicators.

	**3**		**4**		**5**		**6**		**7**		**8**		**9**		***F***	***p***	***η^2^***
**VARIABLES**	***M***	***SD***	***M***	***SD***	***M***	***SD***	***M***	***SD***	***M***	***SD***	***M***	***SD***	***M***	***SD***			
Physical capacities	2	0.001	2	0.001	2	0.001	1.91	0.28	1.79	0.41	1.78	0.41	1.65	0.48	3	0.01	0.05
Mental capacities	2	0.001	2	0.001	2	0.001	1.91	0.28	1.87	0.33	1.79	0.41	1.70	0.46	2.71	0.01	0.05
Psycho-social discomfort	1.74	0.001	1.8	0.001	1.83	0.001	2	0.001	2	0.38	2	0.4	2	0.44	3.09	0.01	0.05
Socio-communicative competence	2	0.001	1.5	0.001	1.33	0.001	1.26	0.5	1	0.47	1	0.47	1	0.44	3.1	0.01	0.05
Number of virtual resources	5	0.001	5	0.001	5	0.001	5	0.001	4.9	0.4	4.72	0.45	4.69	0.91	2.52	0.02	0.04

*Source. Compiled by the authors*.

The collation of the *post-hoc contrasts* revealed statistically significant differences in 120 of the 628 cases analyzed (19.10%), usually upon contrasting studies with a fulfillment of 3, 4, and up to 5 indicators with those which had 6 or more controls.

#### According to age range

The tests for inter-subject effects identified statistically significant differences in the dependent variables relating to socio-communicative competence, professional interest, and level of digital competency of users. Furthermore, as can be seen in Table 5, the studies that bring together a greater number of people are those in which the mean age of the intergenerational groups varies between 50 and 59 years—a group in relation to which the main focus of interventions is social inclusion.

**Table 5:** Statistically significant results taking as grouping variable classification by age range.

**VARIABLES**	**Under 6**		**15–20**		**21–39**		**40–49**		**50–59**		**60–69**		**70–84**		***F***	***p***	***η^2^***
	***M***	***SD***	***M***	***SD***	***M***	***SD***	***M***	***SD***	***M***	***SD***	***M***	***SD***	***M***	***SD***			
Sample	50	0.001	66.54	18.7	57.7	35.81	54.15	55.79	113.3	125.8	105.7	60.91	75.48	26.1	5.61	0.001	0.1
Socio-communicative competence	1	0.001	1	0.5	1.36	0.43	1.75	0.42	2	0.001	1.22	0.001	1	0.001	26.8	0.01	0.4
Digital competence	2	0.001	2	0.001	2	0.21	1.96	0.41	1.95	0.3	1.9	0.001	1.78	0.19	4.03	0.01	0.1
Professional interest	1.23	0.001	1.58	0.001	1.89	0.5	2	0.31	1.78	0.42	1.54	0.51	1	0.42	18.53	0.01	0.3
Recording of sessions	1	0.001	1	0.001	1	0.001	1	0.001	1.4	0.5	1	0.001	1	0.001	27.13	0.001	0.36

*Source. Compiled by the authors*.

When *post-hoc contrasts* between the significant variables obtained in the tests for inter-subject effects by different age ranges are collated, statistically significant differences are observed in 167 of the 738 contrasts analyzed (22.62%). Upon comparing the intergenerational groups whose mean age is between 21 and 39 years with those whose mean age is between 70 and 84 in relation to intervention modality, *p* ≤ 0.03 was obtained. Similarly, based on the field of study that the intervention stems from, we found significant results upon contrasting the groups of 21–39 years vs. 60–69 years (*p* ≤ 0.05).

#### According to the quality of life dimension

The results indicate that with a greater literacy level of the individuals, there is a predominance of interventions related to *material and physical* dimensions, with these focused, as the educational level drops, on improving *social inclusion, interpersonal relations*, and the *self-determination* of the users, and with a focus in the last instance on *personal development* and *emotional well-being* [*M*_*Seld*_ = 1.84; *M*_*EmW*_ = 1.69; *M*_*PhW*_ = 1.93; *M*_*MatW*_ = 2; *M*_*PersDel*_ = 1.76; *M*_*SocIncl*_ = 1.87; *M*_*InterRel*_ = 1.86; *F* = 2.5; *p* = 0.02; η^2^ = 0.04]. It was also confirmed that activities with a focus on *social inclusion* and *interpersonal relations* increased in programs that displayed a greater recording of sessions [*M*_*Seld*_ = 1.03; *M*_*EmW*_ = 1.03; *M*_*PhW*_ = 1; *M*_*MatW*_ = 1; *M*_*PersDel*_ = 1; *M*_*SocIncl*_ = 1.06; *M*_*InterRel*_ = 1.12; *F* = 2.9; *p* = 0.01; η^2^ = 0.05].

With regard to *post-hoc contrasts*, these findings were reinforced and were visible through the significant differences that were found according to the session recording indicator for foci related to *interpersonal relationships* vs. *personal development* (*p* ≤ 0.04). The differences found based on this variable upon comparing the dimensions of *emotional well-being*, which is related to disorders of a mental kind, with the foci of *personal development* (*p* ≤ 0.02), *social inclusion* (*p* ≤ 0.01), *physical well-being* (*p* ≤ 0.009), and *interpersonal relationships* (*p* ≤ 0.001), in which participants predominantly do not have a disorder are also noteworthy. The differences between the *emotional well-being* (40–49) and *physical well-being* (21–39) dimensions are also worth highlighting for the age variable (*p* ≤ 0.05). Statistically significant differences were found in 54 of the 315 analyzed contrasts (17.14%).

#### According to the participants' disorder

Studies whose participants presented a mental disorder (MentD) fulfilled more EBI indicators than those in which subjects suffered from a physical disorder (PhysD) or a combined one (CombD), or those in which subjects had no disorder (AbsD) [*M*_*MentD*_ = 1.8; *M*_*PhysD*_ = 1.76; *M*_*CombD*_ = 1.67; *M*_*AbsD*_ = 1; *F* = 6.51; *p* = 0.001; η^2^ = 0.05].

*Post-hoc contrasts* also displayed statistically significant differences (*p* ≤ 0.007) according to age range for mental disorder (40–49) vs. combined disorder (70–84) and vice versa. In addition, representative results based on certain EBI indicators (such as generalization of the results and follow-up of the interventions) are noteworthy, as are those in relation to the level of commitment to attending the program, membership of an organization, and the literacy level of users.

Finally, other analysis showed statistically significant differences in 51 of the 165 analyzed contrasts (30.90%).

## Discussion

### Summary of main findings

The present systematic review of studies has fulfilled the objective of identifying the main indicators that allow the effectiveness of empirically based intergenerational interventions to be ensured and the hypotheses put forward have been confirmed. We have shown that programs with a greater number of EBI controls have the greatest effectiveness, regardless of the intervention mode employed, and we have demonstrated that this effectiveness is also modulated by other variables such as the participants' disorder, their academic or literacy levels, membership of an organization, and situation of risk of exclusion.

In addition, the need detected in these programs to adapt to the personal circumstances of the users in order to ensure a greater efficiency of the activities justifies the future development and implementation of programs with a marked technological dimension that make use of virtual or combined intervention modes.

Thus, with regard to EBI indicators, it has been shown that if four or more controls were used, then the effect size for the outcomes measured in the interventions is very large, with no statistically significant differences based on the intervention modality used being revealed. As a result, these indicators are indispensable when assessing the actual effect of the interventions and prove their rigor and effectiveness.

It was also confirmed in relation to participants' disorder that as this intensifies, the impact of the interventions grows, with studies whose members have a mental disorder (with whom the dimension of emotional well-being is mainly worked on), thereby fulfilling more EBI indicators than those in which the disorder is of a physical nature.

Moreover, we demonstrated that the lower the level of socio-communicative competence and the worse the physical and mental condition of the subjects, the greater the number of empirically based criteria that programs meet.

Finally, the absence of statistically significant differences concerning the effectiveness of the interventions with regard to the two types of modalities studied justifies the future design and implementation of programs that, while taking care to fulfill EBI controls and remaining being faithful to the objectives and values common to all process of learning and intergenerational education, opt to exploit the benefits offered by digital media and resources to foster the integration of older adults, help the youngest to acquire all kinds of knowledge and skills that contribute to their immediate future, and improve the welfare of families and the community (cfr., Sánchez et al., 2015).

### Limitations

As Kuehne and Kaplan (2001) reported, the nature of intergenerational programs “often results in research and evaluation studies that are descriptive, or limited in the controls they offer when compared with more traditional “experimental” and “control” group, or pre- and post-test designs” (p. 6). This translated into 17 of the 50 studies analyzed in this review featuring a quasi-experimental design of only one intergenerational experimental group with pre-post measures. There was a scarcity of studies that conducted follow-ups, and in most cases, the sample size was a small one. All this means that the results must not be generalized beyond the participants.

In addition, there has been no contribution in discovering which theoretical perspectives have been applied to intergenerational practice in these publications, even though as Kuehne and Melville (2014) assert, “The current state of our literature suggests that we need a broader theoretical base for intergenerational practice that delves deeper into the relational aspects of our work and continues to explore the value and composition of a uniquely intergenerational theory” (p. 337).

Finally, we must indicate that although there is a proliferation of proposals and suggestions for virtual interventions in the field of intergenerational work, these do not have enough empirical validation, and for this reason, they could not be included in the current review, with 7 studies involving the combined intervention modality with consistent reliability found in their place.

## Conclusions

The results derived from this research have identified a series of key elements when it comes to certifying the effectiveness of empirically based intergenerational interventions, as have certain studies in other fields (Scheerens et al., 2013). Our findings suggest future courses of action in the development and implementation of these programs, without prejudice to works that use ethnographic, biographical, life-story, and qualitative interview designs to collect the voices and perceptions of the protagonists of these processes (cfr., Serdio, 2008). The idea is ultimately to incorporate effective and efficient programs that are capable of meeting users' needs, a task that could be facilitated by the use of virtual interventions that are capable of breaking down the communication barriers that exist between the different generations (Bosch and Currin, 2015), to open new pathways for improving young people's empathy toward the elderly (Tabuchi and Miura, 2015) and to appropriately respond to the social isolation that certain age groups experience (Chen and Schulz, 2016). This does not mean that one should ignore the traditional environment and activities that effectively promote individual and cross-age interactions in programs of this type, as is the case of the 5 components identified by Epstein and Boisvert (2006) for this purpose: “A designed space…stocked with materials inviting to both age groups; a consistent daily schedule that allows … cross-age interactions; open-ended activities that emphasize process over product …; the explicit facilitation of cross-age interactions by caregivers; and objective observational assessment to plan activities and share information with families” (p. 87).

It is clear that intergenerational programs cover a wide spectrum of possibilities. Findings suggest intergenerational programs are appropriate and effective for people with dementia (Jarrott and Bruno, 2003, 2007). Moreover, studies have been completed on their role in the development of generativity and psychological well-being in old age and on the possible mediating effects of perceived rejection and respect from younger generations (Tabuchi et al., 2015). Their relevance as examples of high-intensity civic engagement activities, in which older adults serve as mentors and tutors in elementary schools, has been noted (Varma et al., 2015), as has the opportunity that they represent to examine the support that middle-aged adults provide to different generations on a daily basis (Fingerman et al., 2016).

All this provides more than sufficient evidence of the useful role that these programs may play in: reducing negative stereotypes, prejudice, and discrimination associated with older adults and aging (Pillemer et al., 2015; Levy, 2016); implementing any kind of classroom management strategies that improve students' academic, behavioral, social-emotional, and motivational outcomes (Kaskie, 2016; Korpershoek et al., 2016); and expanding the residential, educational, and career options of individuals across the age continuum (Marshall, 2015; Zacher and Schmitt, 2016).

## Author contributions

AC collected the data, searched for references, and prepared the first draft of the article as well as the final version of it. JG designed the study, participated in the collection of the data, and performed the statistical analyses. DP participated in the collection of the data. All authors coded each of the included empirical studies based on the methods used to assess the effectiveness of intergenerational programs. They agreed to be accountable for all aspects of the work in ensuring that questions related to the accuracy or integrity of any part of the work are appropriately investigated and resolved.

### Conflict of interest statement

The authors declare that the research was conducted in the absence of any commercial or financial relationships that could be construed as a potential conflict of interest.

## Abbreviations

EBI: *Empirically based interventions*
EBP: *Evidence-based programs*
LDD: *Learning and developmental disabilities*
Seld: *Self-determination*
EmW: *Emotional well-being*
PhW: *Physical well-being*
MatW: *Material well-bein*
PersDel: *Personal development*
SocIncl: *Social inclusion*
InterRel: *Interpersonal relations*
Med: *Medium*
Lar: *Large*
VerLar: *Very Large*
Fac: Face-to-face
Comb: Combined
MentD: Mental disorder
PhysD: Physical disorder
CombD: Combined disorder
AbsD: Absence of disorder.
